# Glucagon-like Peptide-1 Receptor Agonists in Rheumatoid Arthritis: A Scoping Review of Metabolic, Anti-Inflammatory, and Cardioprotective Effects

**DOI:** 10.3390/jpm16060284

**Published:** 2026-05-26

**Authors:** Simona Buonanno, Carla Gaggiano, Caterina Baldi, Luca Cantarini, Bruno Frediani, Stefano Gentileschi

**Affiliations:** Rheumatology Section, Department of Medical Sciences, Surgery and Neuroscience, Siena University Hospital, University of Siena, Policlinico “Le Scotte”, Viale Mario Bracci 16, 53100 Siena, Italy

**Keywords:** rheumatoid arthritis, glucagon-like peptide-1 receptor agonists, obesity, type 2 diabetes mellitus, cardiovascular risk

## Abstract

Rheumatoid arthritis (RA) is a chronic inflammatory disorder associated with a substantially increased risk of cardiovascular (CV) disease, driven by both persistent systemic inflammation and a high burden of traditional cardiometabolic risk factors. In recent years, glucagon-like peptide-1 receptor agonists (GLP-1RAs), licensed for type 2 diabetes mellitus and obesity, have attracted attention for their broader metabolic and cardiovascular benefits, raising the question of their potential role in RA. This scoping review summarizes current evidence on the impact of GLP-1RAs on RA disease activity, CV comorbidities, and the underlying immuno-metabolic mechanisms. Experimental studies suggest that GLP-1RAs could modulate key inflammatory pathways in synovial cells, reducing pro-inflammatory cytokine production, oxidative stress, and tissue-degrading enzymes, while improving mitochondrial function. Although clinical data remains limited, observational studies report improvements in disease activity, inflammatory markers, and pain in patients with RA treated with GLP-1RAs in addition to immunosuppressive treatment. Extensive evidence from randomized trials in metabolic populations demonstrates that GLP-1RAs improve glycemic control, induce significant weight loss, and reduce modestly but consistently blood pressure and atherogenic lipids, ultimately lowering major CV events and mortality. Although this evidence cannot be directly translated to RA populations, early real-world data specific to the disease suggest similar favorable trends, including reductions in cardiometabolic risk factors and thromboembolic events. Taken together, these findings suggest that GLP-1RAs may offer dual benefits in RA by addressing both metabolic dysfunction and inflammation. However, the current evidence base is heterogeneous and largely non-randomized, underscoring the need for dedicated trials.

## 1. Introduction

Rheumatoid Arthritis (RA) is a chronic systemic inflammatory disease affecting approximately 0.5–1% of the global population [1]. Beyond articular involvement, RA is associated with a substantially increased cardiovascular (CV) risk, estimated to be 1.5–2 times higher than in the general population [2]. This excess risk is multifactorial and arises from a complex interplay between chronic systemic inflammation—promoting accelerated atherosclerosis, endothelial dysfunction, oxidative stress, and lipid alterations, including the “lipid paradox” [3,4,5,6]—and traditional CV risk factors such as hypertension, diabetes mellitus, dyslipidemia, obesity, and smoking. In addition, RA-specific features, including activity, autoantibody status, and disease duration, further contribute to CV risk [7]. Current guidelines recommend periodic CV risk assessment using general population tools (e.g., Systematic Coronary Risk Evaluation [SCORE], QResearch Cardiovascular Risk Algorithm [QRISK]), although their accuracy in RA is limited and may require adjunctive approaches such as carotid ultrasound for improved stratification [4,8]. Optimal management therefore requires both tight control of inflammatory activity and aggressive treatment of modifiable CV risk factors.

Glucagon-like peptide-1 receptor agonists (GLP-1RAs) are a class of agents targeting incretin pathways. GLP-1 is a pleiotropic hormone with widespread receptor distribution across multiple tissues, enabling broad systemic effects [9]. These medications have transformed the management of type 2 diabetes mellitus (T2DM) by improving glycemic control through glucose-dependent insulin secretion and suppression of glucagon release. Several GLP-1RAs, including liraglutide, semaglutide, and tirzepatide, are also approved for obesity treatment, promoting weight loss (WL) via increased satiety and reduced caloric intake. Importantly, their use is associated with improved CV and renal outcomes, as well as reduced overall mortality [10,11,12,13]. Recent advances have expanded this therapeutic class through the development of dual (GLP-1/amylin) and triple (GLP-1/Gastric Inhibitory Polypeptide [GIP]/glucagon) receptor agonists designed to target additional entero-pancreatic pathways and exploit complementary, potentially synergistic mechanisms, thereby further improving metabolic efficacy [14].

In this context, this scoping review aims to provide an overview of the current evidence regarding the use of GLP-1RAs in patients with RA, with specific regard to their impact on both CV comorbidities and potential effects on disease activity. The review will also investigate the underlying immuno-metabolic mechanisms through which GLP-1RAs may exert metabolic and anti-inflammatory benefits.

## 2. Methods

Our methodological approach adhered to the PRISMA 2018 Statement extensions for scoping reviews. The review was not registered in PROSPERO or in any other international database.

### 2.1. Information Sources

Comprehensive literature research was conducted in PubMed, MEDLINE, Scopus, Embase, and Google Scholar covering their respective inceptions to March 2026.

### 2.2. Inclusion Criteria

The review included preclinical/basic research (in vitro/mechanistic studies) and clinical studies (observational studies and randomized controlled trials [RCTs]). Evidence synthesis studies (systematic reviews and meta-analyses) were included as a source of high-level evidence to highlight consistent trends and existing knowledge gaps. Recent conference abstracts were included to capture the full breadth of available evidence on GLP-1RA use in rheumatoid arthritis, an emerging area of research with findings not yet available as full-text articles.

### 2.3. Eligibility Criteria

Eligibility was determined using the Population–Concept–Context framework:

- Population: people affected by RA (direct evidence to the topic); individuals with overweight/obesity or T2DM (indirect evidence to the topic).
- Concept: effects of GLP-1RAs on RA disease activity, risk of RA onset, and cardiovascular (CV) comorbidity outcomes, including evidence derived from non-RA populations when relevant to CV effects.
- Context: preclinical and clinical settings, including in vitro studies, observational studies, randomized controlled trials, and evidence synthesis studies, with no geographical restrictions.

### 2.4. Search Strategy

Search terms combined free-text keywords and MeSH terms related to rheumatoid arthritis, GLP-1RAs, obesity, T2DM, and CV risk factors (blood pressure [BP], lipid profile, atherosclerosis, major adverse CV events [MACEs]). The search strategy was first developed for PubMed and then adapted for additional databases. Complete search strings are reported in Supplementary Materials S1.

**Figure 1:**
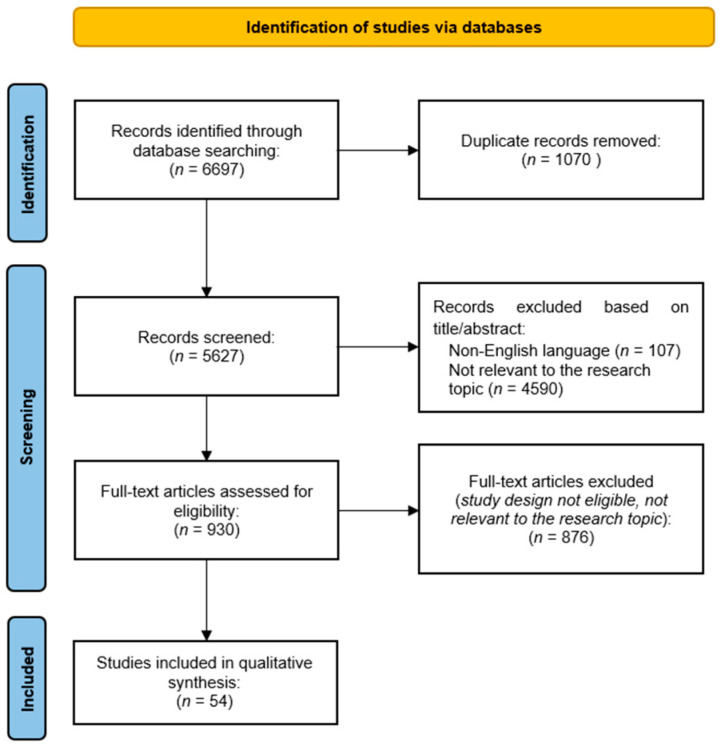
PRISMA flow diagram of the study selection process.

Screening of the studies, initially by language (English), titles and abstracts and subsequently by full-text review (Figure 1), was based on their relevance to four predefined topics:

1. Potential effects of GLP-1RAs on RA disease activity: preclinical and clinical evidence;
2. Potential impact of GLP-1RAs on the risk of developing RA;
3. Efficacy of GLP-1RA on RA CV comorbidities;
4. CV Outcomes Associated with GLP-1RA treatment from RA trials.

### 2.5. Selection of Sources and Data Charting

Data extraction utilized a standardized form capturing author, year, country, study design and population, key outcomes and results, and main conclusions.

Studies were excluded if they: (1) did not evaluate GLP-1RAs as an intervention or exposure; (2) did not include RA, T2DM or obese/overweight populations; (3) were not available in English; (4) were editorials or commentaries without original data; or (5) full text could not be retrieved.

All citations retrieved from the database search were imported into Microsoft Excel for deduplication and preliminary screening. Two reviewers (S.B. and C.G.) independently screened titles and abstracts according to the predefined eligibility criteria. Full texts of potentially relevant studies were then retrieved and assessed independently by the same reviewers for final inclusion. Any disagreements were resolved through discussion until a consensus was reached.

### 2.6. Data Analysis and Presentation

Data were synthesized narratively and supplemented with tables and figures. In line with scoping review guidelines, a formal risk of bias assessment was not conducted; however, the methodological limitations of the included studies were appraised narratively.

## 3. Results

### 3.1. Potential Effects of GLP-1RAs on RA Disease Activity

A summary of preclinical and clinical studies evaluating the potential effects of GLP-1RAs in RA patients is provided in Table 1 and Table 2.

#### 3.1.1. Preclinical Studies

Three studies have investigated the immunomodulatory effects of GLP-1RAs by using Tumor Necrosis Factor (TNF) or Interleukin (IL)-1β to induce inflammatory responses in fibroblast-like synoviocytes (FLSs) [15,16,17]. Fibroblast-like synoviocytes are central mediators of synovial inflammation and joint destruction in RA, making them widely used in vitro models to evaluate the anti-inflammatory and cartilage-protective potential of GLP-1RAs. Across these studies, critical effects of GLP-1RAs on RA-FLSs have been observed. Dulaglutide, exenatide, and lixisenatide restored mitochondrial function, as evidenced by improved mitochondrial membrane potential and enhanced enzymatic activity [15,16,17]. Oxidative stress was significantly reduced, with decreases in reactive oxygen species (ROS) and Nicotinamide Adenine Dinucleotide Phosphate (NADPH) Oxidase 4 (NOX-4) reported for all three compounds, with an additional reduction in 4-Hydroxynonenal (4-HNE) noted for lixisenatide [15,16,17]. Furthermore, all agents downregulated key pro-inflammatory mediators, including IL-1β, IL-6, TNF, Monocyte Chemoattractant Protein-1 (MCP-1), and High Mobility Group Box (HMGB-)1, indicating a broad anti-inflammatory effect [15,16,17]. Matrix metalloproteinases (MMP) (MMP-1, MMP-3, MMP-13), which contribute to cartilage degradation and synovial pannus formation, were also suppressed across all studies, indicating a potential chondroprotective effect [15,16,17]. Mechanistically, all three GLP-1RAs inhibited nuclear factor kappa B (NF-κB) activation, while dulaglutide and lixisenatide additionally inhibited c-Jun N-terminal Kinase (JNK) phosphorylation and activator Protein 1 (AP-1) signaling, and exenatide acted through p38/Mitogen-Activated Protein Kinase (MAPK) in addition to NF-κB blockade [15,16,17]. Despite these common features, some differences between compounds were observed. Lixisenatide exhibited anti-apoptotic effects, enhancing FLS survival while downregulating pro-inflammatory cytokine production and inflammatory cellular signaling pathways [17], whereas dulaglutide and exenatide focused primarily on restoring mitochondrial function and reducing oxidative stress [15,17]. In addition, exenatide displayed dose-dependent effects, with near-complete normalization of inflammatory and mitochondrial parameters at the higher concentration (20 nM) [16].

#### 3.1.2. Observational Retrospective and Prospective Studies

Evidence on the use of GLP-1RAs in RA remains limited, with most available data deriving from small, prospective cohorts or retrospective real-world analyses.

Sullivan et al. prospectively evaluated the disease course of 15 patients with inadequately controlled T2DM and concomitant active inflammatory arthritis (11 RA, 4 Psoriatic arthritis [PsA]) over 24 weeks when liraglutide (1.2 mg s.c. daily) was added to background immunosuppressive treatment. Among participants, 9/15 achieved a European League Against Rheumatism (EULAR) Disease Activity Score 28 (DAS28) response, with body weight, Glycated Hemoglobin (HbA1c), and swollen joint count significantly improved in responders; non-responders showed no meaningful changes. Weight loss and HbA1C reduction were positively associated with DAS28 response, suggesting that metabolic improvements may contribute to anti-inflammatory effects, although the study was underpowered for RA-specific conclusions [18].

Similar findings were reported in an observational cohort in which 30 patients with RA, obesity, and T2DM received GLP-1RAs for six months without concomitant conventional (c-) or biologic (b-) Disease-Modifying Anti-Rheumatic Drugs (DMARDs) or glucocorticoids. Patients experienced an average of 10% WL, improved morning stiffness and joint swelling, decreased finger pain, and reductions in C-Reactive Protein (CRP) and Erythrocyte Sedimentation Rate (ESR).

Stipho et al., using real-world clinical data network for research TriNetX, reported that semaglutide reduced the risk of RA flare-ups in 12,139 patients, with lower rates of synovitis, stiffness, swelling, and pain at 30 days, 90 days, and one year. These results suggest a rapid and sustained benefit on joint outcomes, although causality cannot be inferred due to the retrospective design and lack of dosing information [20].

Similarly, Dente et al. examined 152 RA patients treated with semaglutide or tirzepatide over one year, reporting significant reductions in weight, Body Mass Index (BMI), ESR, CRP, pain scores, and lipid parameters. While composite RA disease activity measures were incomplete, the study suggests that anti-obesity medications may be associated with improvements in both metabolic and inflammatory parameters in patients with RA [21].

In a single-center retrospective study, 173 RA patients treated with semaglutide or tirzepatide were compared to 42 controls who were prescribed but did not take GLP-1RAs. Baseline characteristics were broadly comparable between groups in terms of BMI, disease activity, and background antirheumatic therapy, although the treated group exhibited a higher burden of cardiometabolic comorbidities. Patients were followed for up to 12 months with longitudinal assessments of clinician-derived RA disease activity (remission, mild, moderate, or severe), pain visual analogue scale (VAS), body weight, HbA1c, lipid profile, and inflammatory markers, alongside treatment changes and adverse events. Treated patients demonstrated significant reductions in RA disease activity, pain measured by VAS, body weight, total cholesterol, HbA1c, ESR, CRP, Low-Density Lipoprotein Cholesterol (LDL-C), and triglycerides. Notably, decreases in acute-phase reactants and reduction in VAS pain score were not correlated with WL, indicating potential weight-independent anti-inflammatory and analgesic effects. Sensitivity analyses adjusting for T2DM, hypertension, autoantibody profile, and ethnic background confirmed the robustness of these findings. However, the retrospective single-center design, limited sample size, potential selection bias, and lack of standardized disease activity measures constrain causal inference [22].

### 3.2. Influence of GLP-1RAs on the Risk of Developing RA

Recent real-world studies have investigated the relationship between GLP-1RAs and the incidence of autoimmune diseases, including RA.

A large retrospective TriNetX cohort study evaluated patients with T2DM or obesity receiving GLP-1RAs compared with those not treated with these agents. GLP-1RA use was associated with lower 5-year incidence of several systemic diseases, including systemic lupus erythematosus, systemic sclerosis, intestinal bowel diseases, and osteoporosis. With respect to RA, GLP-1RA therapy was associated with a significantly reduced incidence in obese individuals without T2DM, supporting a potential role of immunometabolic mechanisms in RA development; no significant differences were observed among patients with T2DM [23].

Conversely, a population-based case–control study investigated the association between GLP-1RA (liraglutide, semaglutide, and dulaglutide) exposure and new-onset RA in 4535 RA cases matched to 22,675 unexposed controls. After adjusting for age, BMI, smoking, T2DM, and socioeconomic status, liraglutide and semaglutide use was significantly associated with new onset RA. Notably, this association was more pronounced with shorter treatment durations (≤6 months) and attenuated with longer exposure, suggesting a potential early immune activation followed by risk mitigation, possibly mediated by WL, improved insulin sensitivity, and reduced systemic inflammation. These findings raise the hypothesis that GLP-1RAs may exert complex immunometabolic effects, including both anti-inflammatory actions (e.g., TNF, IL-6 suppression, NF-κB inhibition) and transient pro-inflammatory responses through innate immune pathways, which could contribute to RA onset in some individuals. However, the observational nature of the study precludes any mechanistic or causal conclusions regarding their role in RA onset [24].

### 3.3. Impact of GLP-1RA on RA Comorbidities

A summary of major RCTs and meta-analyses assessing GLP-1RAs effects on T2DM, obesity, and CV comorbidities frequently observed in RA patients is available in Supplementary Tables S2–S4.

The CV and metabolic effects of GLP-1RAs have been extensively investigated through high-quality evidence synthesis in patients with T2DM and obesity. Landmark clinical trials, including the LEADER, SUSTAIN-6, and REWIND, consistently show that GLP-1RAs lower MACEs, CV and all-cause mortality, and concurrently improve cardiometabolic outcomes such as body weight, glycemic control, BP, postprandial lipid levels, and inflammatory biomarkers [25,26]. Mechanistic evidence suggests that these benefits may result from both indirect effects via metabolic improvements and potential direct actions on the heart and vasculature, where GLP-1 receptors are expressed at low levels [26].

#### 3.3.1. Type 2 Diabetes Mellitus

Acting as analogs of endogenous GLP-1, GLP-1-based therapies have emerged as one of the most effective therapeutic options for managing T2DM due to their multifaceted effects on glucose metabolism. They improve glycemic control by enhancing glucose-dependent insulin secretion, suppressing glucagon release, slowing gastric emptying, and modulating appetite [14]. GLP-1RAs, including exenatide, dulaglutide, and lixisenatide, have demonstrated high efficacy in improving glycemic control by markedly reducing postprandial glucose and HbA1c levels. More recently, dual and triple agonists—particularly tirzepatide—have shown even greater effectiveness, achieving superior reductions in HbA1c and fasting blood glucose, supported by robust evidence [27].

In DURATION trials, exenatide lowers HbA1c and postprandial glucose, with the once-weekly regimen demonstrating greater efficacy compared to the twice-daily option [28].

Dulaglutide demonstrated superior glycemic control compared to exenatide in the AWARD-1 study, with greater HbA1c reductions [29], while it was noninferior to liraglutide in HbA1c reduction (AWARD-6) [30].

Liraglutide was more effective than lixisenatide as an add-on to metformin in improving glycemic control, producing significantly greater reductions in HbA1c and fasting plasma glucose than lixisenatide, with more patients achieving glycemic targets. Lixisenatide showed a stronger effect on postprandial glucose excursions following the administered meal, while overall daily glycemic control favored liraglutide [31].

In the SUSTAIN 1, 3, 7, and 10 trials, once-weekly subcutaneous semaglutide consistently reduced HbA1c and improved fasting plasma glucose compared with other long-acting GLP-1RAs (weekly exenatide, dulaglutide, liraglutide), with these effects being dose-dependent [32,33,34,35]. The PIONEER trials consistently showed that oral semaglutide provides robust, dose-dependent improvements in glycemic control in adults with T2DM. Its efficacy was comparable or superior to other GLP-1RAs, including liraglutide and dulaglutide, and clearly exceeded placebo, demonstrating its effectiveness across different populations and treatment regimens [36,37,38,39].

The SURPASS trials consistently demonstrated that tirzepatide provides substantial, dose-dependent reductions in HbA1c in patients with T2DM. Across multiple studies, tirzepatide showed superior glycemic and weight-lowering effects compared with placebo, basal insulin, or prandial insulin [40,41,42,43]. In SURMOUNT-1 trial, tirzepatide markedly lowered the risk of progression from prediabetes to T2DM over long-term treatment [44].

The phase 2 trial of orforglipron, an oral non-peptide GLP-1RA, demonstrated substantial, dose-dependent reductions in HbA1c and body weight in adults with T2DM. Efficacy exceeded that of placebo and was greater than dulaglutide, suggesting the drug as a convenient oral alternative to injectable GLP-1RA therapies for achieving glycemic and weight-loss goals [45].

The CagriSema trial showed that the combination of semaglutide and cagrilintide produced greater HbA1c reductions than either agent alone in adults with T2DM and elevated BMI. The dual therapy demonstrated enhanced, dose-dependent glycemic efficacy over 32 weeks, highlighting the benefits of combining GLP-1 and amylin receptor agonism [46].

A randomized double-blind phase 2 trial demonstrated that retatrutide produces substantial, dose-dependent reductions in HbA1c in adults with T2DM and elevated BMI. Higher weekly doses of retatrutide achieved greater glycemic improvements compared with dulaglutide or placebo [47].

#### 3.3.2. Obesity

Evidence supporting the use of GLP-1RAs for weight management in obesity is robust and mainly derived from large RCTs and meta-analyses. Two recent systematic reviews consistently demonstrate that GLP-1RAs—including semaglutide, liraglutide, exenatide, and efpeglenatide—produce significant reductions in body weight, BMI, and waist circumference in individuals with overweight or obesity, both with and without T2DM. Across analyses, the magnitude of the effect varies among different agents and study populations; semaglutide emerged as the most effective agent for WL, with sustained benefits observed with longer treatment duration [48,49].

In the DURATION-1 trial, once-weekly exenatide produced sustained WL of 4.1–4.5 kg over 52 weeks in patients with T2DM, accompanied by improved glycemic control, with 77–79% of participants achieving concurrent reductions in HbA1c and body weight [50]. Compared with newer agents, the magnitude of WL was modest but durable.

In a 56-week placebo-controlled trial in adults with overweight or obesity without T2DM, daily liraglutide 3.0 mg induced significantly greater WL than placebo. A higher proportion of participants achieved clinically meaningful WL, alongside improvements in cardiometabolic risk factors and glycemic parameters [51]. These findings indicate a stronger anti-obesity effect than earlier GLP-1RAs such as exenatide.

Multiple trials have shown that weekly semaglutide exhibits strong WL efficacy. Treatment resulted in substantial WL compared with placebo, with most participants achieving clinically meaningful WL and improvements in cardiometabolic risk factors [52]. Subsequent studies demonstrated dose-dependent effects of semaglutide, with higher doses (7.2 mg) producing greater WL in individuals with obesity without T2DM [53]. Similarly, in the STEP UP T2D trial, the 7.2 mg dose achieved a mean WL of 13.2% in patients with obesity and T2DM, alongside improvements in HbA1c and waist circumference [54].

Tirzepatide has demonstrated pronounced metabolic benefits. In the SURMOUNT 2 trial, treatment in patients with obesity and T2DM led to substantial WL compared with placebo [55]. Those findings were consistent with the SURPASS 1 trial, where tirzepatide also produced dose-dependent WL and marked reductions in HbA1c in patients with T2DM [43].

The phase 2 trial of orforglipron demonstrated substantial, dose-dependent WL in adults with T2DM, with higher doses producing greater WL compared with placebo and dulaglutide [45].

In a phase 2 trial of adults with T2DM and overweight/obesity, treatment with CagriSema 2.4 mg resulted in greater WL compared with cagrilintide 2.4 mg or semaglutide 2.4 mg alone [46].

Phase 2 studies have demonstrated that retatrutide induces substantial, dose-dependent WL in adults with obesity, both with and without T2DM. In participants without T2DM, once-weekly administration resulted in WL of up to 24.2%, with 36–48% of those receiving 8–12 mg achieving ≥25% WL, with effects being particularly pronounced in individuals with BMI ≥ 35 kg/m^2^ and in women [56]. Similarly, in adults with T2DM, retatrutide produced progressive WL, ranging from 3.2% at 0.5 mg to nearly 17% at 12 mg, far exceeding reductions observed with placebo (2–3%) or 1.5 mg dulaglutide (2%) [47].

#### 3.3.3. Blood Pressure

GLP-1RAs have been consistently associated with modest reductions in BP in individuals with overweight or obese. Across recent meta-analyses, the overall magnitude of BP reduction appears relatively consistent, with systolic (s)BP decreasing by approximately 3–4 mmHg and diastolic (d)BP by about 1 mmHg 57–59. In a meta-analysis of 30 RCTs, GLP-1RAs (semaglutide, liraglutide, and exenatide) reduced sBP and dBP, with semaglutide showing the numerically largest sBP reduction, followed by liraglutide and exenatide; nevertheless, subgroup analyses did not demonstrate significant differences between agents, supporting a class-consistent effect [57]. Similar findings were reported in a larger systematic review and meta-analysis of 85 RCTs, which found reductions of −3.4/−0.9 mmHg (sBP/dBP) with GLP-1RAs and identified greater WL as a key factor of BP reduction [58]. Importantly, both this analysis and a 2025 network meta-analysis of 75 RCTs highlighted differences across incretin-based therapies, with larger BP reductions observed for multi-receptor agonists. Dual GIP/GLP-1 agonists were associated with reductions of approximately −5 mmHg sBP, whereas triple agonists showed the greatest effects, with sBP reductions approaching −6 to −7 mmHg [58,59].

#### 3.3.4. Lipid Profile

GLP-1RAs have also been evaluated for their effects on lipid metabolism. Overall, available evidence suggests modest but consistent improvements in lipid parameters, particularly low-density lipoprotein-cholesterol (LDL-C), total cholesterol (TC), and triglycerides (TG). An early systematic review and network meta-analysis of 35 RCTs in patients with T2DM showed that all GLP-1RAs significantly reduced LDL-C compared to placebo, insulin, and thiazolidinediones. Liraglutide and taspoglutide also significantly lowered TC, while TG reductions were mainly observed with liraglutide and taspoglutide. In contrast, high-density lipoprotein (HDL)-C did not improve, with small decreases reported for some agents [60]. More recent analyses confirm these findings while highlighting differences across incretin-based therapies. A 2025 network meta-analysis reported that GLP-1RAs significantly reduced TC and LDL-C compared with placebo and Sodium-Glucose Co-Transporter 2 (SGLT2) inhibitors, while dual GIP/GLP-1 agonists produced larger reductions, including LDL-C, TG and TC [61]. Similarly, a 2023 network meta-analysis including 15 GLP-1RAs found that semaglutide significantly reduced LDL-C and TC, whereas tirzepatide and ITCA 650 (implantable exenatide-delivery device) produced marked TG reductions. Notably, newer dual and multi-agonists (e.g., tirzepatide, mazdutide, CagriSema, and retatrutide) demonstrated stronger metabolic effects overall, including more pronounced lipid improvements [27].

#### 3.3.5. Atherosclerosis and MACEs

Findings from preclinical models, observational studies, and meta-analyses of RCTs suggest a potential modulatory role of GLP-1RAs in atherogenic signaling, potentially translating into clinically relevant CV benefits. Preclinical studies are consistent with direct anti-atherosclerotic effects of GLP-1RAs. In apolipoprotein (Apo)E−/− and LDLr−/− mouse models, treatment with liraglutide or semaglutide significantly reduced aortic plaque formation and intimal thickening, effects partly independent of weight and lipid changes. Transcriptomic analyses showed down-regulation of inflammatory pathways involved in leukocyte recruitment and vascular inflammation, supporting a mechanism mediated by anti-inflammatory and vascular protective actions [62]. In humans, observational data provides indirect evidence linking endogenous GLP-1 levels to coronary atherosclerosis. In a cohort of 303 patients undergoing coronary computed tomography angiography, higher circulating GLP-1 concentrations were positively associated with total coronary plaque burden, independently of traditional CV risk factors. Although causality cannot be inferred, these findings suggest a possible involvement of GLP-1 pathways in the human atherosclerotic process [63]. Clinical evidence also indicates favorable vascular effects, although structural outcomes remain less clear. A meta-analysis evaluating GLP-1-based therapies (including GLP-1RAs and dipeptidyl peptidase-4 [DPP-4] inhibitors) in patients with T2DM found no significant long-term improvement in flow-mediated dilation (FMD) or carotid intima-media thickness. However, acute studies demonstrated significant increases in FMD, indicating short-term enhancement of endothelial function. In addition, several circulating markers associated with atherogenesis—including high-sensitivity CRP, plasminogen activator inhibitor-1, and B-type natriuretic peptide—were significantly reduced, along with modest improvements in TC, LDL-C, and TG [64]. Consistent with these mechanistic and vascular effects, cardiovascular outcome trials (CVOTs) have demonstrated that GLP-1RAs confer significant CV protection in patients with T2DM, particularly those at high CV risk [26]. A large meta-analysis including eight major CVOTs (ELIXA, LEADER, SUSTAIN-6, EXSCEL, Harmony Outcomes, REWIND, PIONEER 6, and AMPLITUDE-O) showed that GLP-1RAs reduced MACEs by 14%, with consistent effects across dosing regimens, structural classes, and baseline risk profiles. Reductions were observed across individual components, including CV death, myocardial infarction, and stroke [65]. More recent evidence confirms these findings in broader populations. A systematic review and meta-analysis of 21 RCTs including 99,599 patients reported that GLP-1RAs significantly reduced all-cause mortality, CV death, and MACEs, over a mean follow-up of 2.4 years. Significant reductions were also observed for myocardial infarction and heart failure hospitalization (both −15%), whereas stroke did not significantly decrease. Cardiovascular benefits were consistent across subgroups with or without T2DM, obesity, chronic kidney disease, or heart failure, although obese individuals experienced a greater reduction in heart failure hospitalization. Agent-specific analyses suggested generally consistent MACE reductions with liraglutide, semaglutide, and dulaglutide, with only minor heterogeneity between drugs [25].

### 3.4. CV Outcomes in RA Trials

Evidence on the CV impact of GLP-1RAs in RA is still limited but is increasingly emerging from observational studies (Table 3).

In a retrospective cohort of patients with RA and overweight/obesity, GLP-1RA treatment (semaglutide or tirzepatide) resulted in significant reductions in body weight, BMI, HbA1c, and TC over 12 months. These changes were accompanied by decreases in LDL-C, TG, and systemic inflammatory markers (ESR and CRP), while BP changes were modest. Although MACEs were not directly assessed, these metabolic and inflammatory improvements may be consistent with a potentially more favorable CV risk profile [22].

Similar findings were also reported as a conference abstract by the same authors evaluating semaglutide or tirzepatide in patients with RA, showing improvements in body weight, BMI, lipid parameters, and inflammatory markers [21]. In addition, Sullivan et al. reported significant reductions in both body weight and HbA1c in a small prospective cohort of patients with inflammatory arthritis and concomitant T2DM who achieved a clinical DAS28 response [18].

Larger retrospective analyses focusing on clinical outcomes have reported reductions in thromboembolic events. In a global propensity score-matched cohort of patients with RA and T2DM, GLP-1RAs use was associated with a significantly lower risk of overall thromboembolic events compared with DPP-4 inhibitors. Risk reductions were observed for myocardial infarction, stroke, and deep vein thrombosis, as well as lower risks of both arterial and venous thrombosis and reduced all-cause mortality [66].

Similarly, among RA patients treated with Janus Kinase inhibitors, the addition of GLP-1RAs was associated with lower rates of arterial CV events and acute coronary syndromes, as well as fewer venous thromboembolic events. However, reductions in stroke and peripheral artery thrombosis did not reach statistical significance, and overall survival was comparable between groups [67]. Further evidence derives from large-scale retrospective database analyses. Loizidis et al. examined a cohort of 2698 obese patients with RA without T2DM who initiated treatment with liraglutide, semaglutide, or tirzepatide, and compared them with matched RA controls. At one year, GLP-1RA therapy was associated with a lower rate of all-cause mortality and a reduction in new-onset MACEs, primarily attributable to a decreased incidence of heart failure [68].

## 4. Discussion

### 4.1. Targeting Cardiometabolic Comorbidities in RA Patients

RA is characterized by a high cardiometabolic burden, with a markedly increased risk of CV disease and mortality compared with the general population [6,69,70]. While traditional risk factors substantially contribute, chronic systemic inflammation and disease-specific features play a central pathogenic role, supporting the concept of RA as an independent CV risk condition comparable to T2DM [5,71,72]. Both traditional risk factors and disease-related characteristics have been associated with increased CV events in RA populations [7,73]. Moreover, insulin resistance, highly prevalent in RA, correlates with inflammatory mediators such as IL-6, TNF, and CRP, and appears to be linked to disease activity even after accounting for glucocorticoid exposure [74,75,76,77,78]. Consequently, current recommendations emphasize a comprehensive approach to CV prevention that includes aggressive control of systemic inflammation alongside systematic screening and management of modifiable cardiometabolic risk factors [79]. Given this elevated cardiometabolic burden and the frequent presence of insulin resistance in RA, interventions that improve both metabolic and CV outcomes, such as GLP-1RAs, may hold particular clinical relevance in this population.

The cardioprotective effects of GLP-1RAs have been robustly demonstrated in the general population with T2DM or obesity through large randomized CVOTs and meta-analyses. Large trials consistently demonstrate improvements in cardiometabolic risk factors, including body weight, glycemic control, BP, lipid parameters, and systemic inflammation, supporting both indirect metabolic and potential direct vascular protective mechanisms of GLP-1 signaling [26,27,28,29,30,31,32,33,34,35,36,37,38,39,40,41,42,43,44,45,46,47,48,49,50,51,52,53,54,55,56,57,58,59,60,61,62,63,64,65,66,67,68,69,70,71,72,73,74,75,76,77,78,79,80]. However, the extent to which these benefits translate to RA populations remains uncertain and largely inferential since these findings are not specific to RA populations and should be interpreted as indirect evidence. Moreover, most CVOTs and large meta-analyses do not provide detailed information regarding the presence of comorbid RA among enrolled participants. Consequently, it is not possible to determine whether patients with RA were included in these populations or to extrapolate RA-specific cardiometabolic and CV outcomes from these studies.

In contrast, studies conducted specifically in RA populations are predominantly retrospective and observational, resulting in a lower level of clinical evidence. Available analyses suggest that treatment with GLP-1RAs may improve cardiometabolic parameters, potentially contributing to a reduction in CV risk in patients with RA [21,22]. Both Dente et al. and Kellner et al. demonstrated significant improvements in glycemic control, body weight, and lipid profile in RA patients treated with GLP-1RAs, supporting their potential role in managing cardiometabolic comorbidities in this population [21,22]. Additional real-world studies report associations between GLP-1RA exposure and lower rates of arterial and venous thromboembolic events compared with other antidiabetic therapies or background treatments [66,67]. Within this framework, evidence from large retrospective database studies offers further, though indirect, indications of a cardioprotective effect, pointing toward lower mortality and CV event rates, with benefits appearing to be largely influenced by improvements in heart failure outcomes [68]. Nevertheless, heterogeneity in study populations, designs, and endpoints, together with the absence of randomized CVOTs in RA, limits causal inference and highlights the need for prospective controlled studies to confirm these preliminary cardiometabolic benefits in this high-risk population.

### 4.2. Immunometabolic Effects of GLP-1RAs and Potential Implications for RA Pathogenesis

This review highlights emerging evidence that GLP-1RAs may modulate RA pathogenesis and disease activity through interconnected metabolic and immune mechanisms, providing a framework to interpret both clinical observations and experimental findings. Observational data indicates that metabolic status may modulate this relationship: the reduced RA incidence observed in obese individuals treated with GLP-1RAs suggests that WL, improved insulin sensitivity, and reduction in adipose-derived inflammatory mediators may attenuate systemic inflammation and potentially lower autoimmune risk [23]. In contrast, the transient increase in RA incidence reported by Israel et al. during the first months of therapy may reflect early immune activation, possibly mediated by innate immune pathways including Toll-like receptor signaling, NF-κB activation, and transient cytokine release [24]. This apparent discrepancy may represent a biphasic effect of GLP-1RAs, in which initial pro-inflammatory signaling precedes the longer-term metabolic and anti-inflammatory benefits associated with sustained therapy, such as WL and improved glycemic control. These findings underscore the complexity of GLP-1RA immunomodulation and highlight the need for careful monitoring during early treatment, as well as for further mechanistic and longitudinal studies to clarify their dual immune-metabolic effects.

### 4.3. Mechanistic Evidence from In Vitro Studies

Mechanistic in vitro studies in RA-FLSs further support a direct anti-inflammatory role of GLP-1RAs, demonstrating suppression of key cytokines (IL-1β, IL-6, TNF), inhibition of NF-κB and MAPK signaling, and reduced expression of MMP. Additionally, the restoration of mitochondrial function and reduction in oxidative stress observed in these models suggest a link between metabolic regulation and inflammatory control [15,16,17]. The apparent differences in the cellular effects reported across studies evaluating lixisenatide, dulaglutide, and exenatide may be partly explained by methodological heterogeneity rather than true compound-specific mechanistic divergence. Indeed, these studies differ in the inflammatory stimulus used (TNF-α for Dulaglutide and Exenatide versus IL-1β for lixisenatide), drug exposure duration and concentration, and primary experimental endpoints. In particular, while some analyses focused on inflammatory signaling pathways [15], others primarily assessed mitochondrial function or cell survival [16,17]. Therefore, the observed variability is likely driven by differences in experimental design and readouts, rather than fundamentally distinct mechanisms among GLP-1RAs. Together, these findings support a model in which GLP-1RAs may exert both indirect metabolic effects—through WL and improved metabolic homeostasis—and direct immunomodulatory actions within the synovial microenvironment, potentially influencing RA onset and inflammatory activity.

### 4.4. Clinical Implications for Disease Activity and Inflammatory Outcomes

The mechanistic findings at the cellular level may help explain the clinical improvements reported in observational studies conducted in patients with RA treated with GLP-1RAs. In a prospective cohort, liraglutide treatment was associated with a EULAR DAS28 response in the majority of patients, with WL correlating with clinical improvement [18]. Similarly, reductions in inflammatory markers and joint symptoms were observed among RA patients with obesity and T2DM treated with GLP-1RAs, suggesting an overall improvement in clinical and inflammatory status in parallel with weight loss [19].

**Figure 2:**
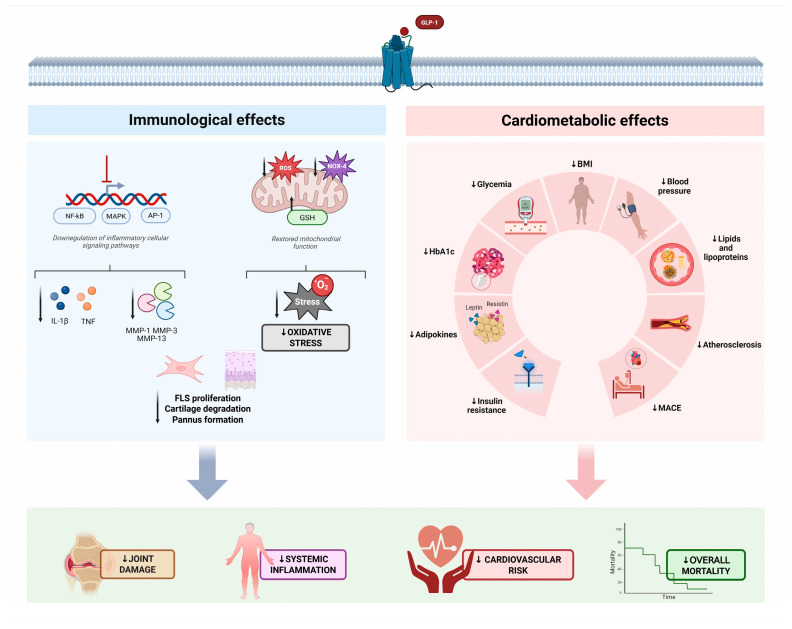
Potential immunological and cardiometabolic effects of glucagon-like peptide-1 receptor agonists (GLP-1RAs) in rheumatoid arthritis (RA). RA is associated with increased cardiovascular risk driven by both traditional factors and chronic systemic inflammation. GLP-1RAs may improve cardiometabolic profiles through weight loss, enhanced insulin sensitivity, and better glycemic and lipid control, while also exerting direct anti-inflammatory effects. These include inhibition of pro-inflammatory cytokines, suppression of nuclear factor kappa B (NF-κB) and Mitogen-Activated Protein Kinase (MAPK) signaling, and reduction in oxidative stress, potentially contributing to decreased disease activity and CV risk. Created in BioRender. Buonanno, S. (2026) https://BioRender.com/lcs3fm6 (accessed on 10 April 2026). Abbreviations: AP-1: activator Protein 1; BMI: body mass index; FLSs: fibroblast-like synoviocytes; GLP-1: glucagon-like peptide-1; GSH: glutathione; HbA1c: glycated hemoglobin; IL: interleukin; MACEs: major adverse cardiovascular events; MAPK: Mitogen-Activated Protein Kinase; MMP: matrix metalloproteinases; NF-κB: nuclear factor kappa B; NOX-4: Nicotinamide Adenine Dinucleotide Phosphate (NADPH) Oxidase 4; ROS: reactive oxygen species; TNF: tumor necrosis factor.

Larger real-world analyses further suggest potential benefits, including reduced RA flare rates with semaglutide [20] and improvements in inflammatory markers and pain scores in patients treated with semaglutide or tirzepatide [21,22]. Notably, some studies reported reductions in inflammatory markers independent of WL, supporting a preliminary hypothesis that GLP-1RAs may exert direct immunomodulatory effects beyond their metabolic benefits. Overall, although current evidence is largely observational, these findings suggest that GLP-1RAs may represent a promising adjunctive strategy to improve inflammatory and metabolic outcomes in patients with RA (Figure 2).

### 4.5. Strengths and Limitations of Current Evidence

Despite the growing interest in the potential role of GLP-1 RAs in RA, the current body of evidence remains limited and should be interpreted with caution. Most available data derive from retrospective cohort studies, small observational studies, and conference abstracts, while RCTs specifically evaluating inflammatory and rheumatologic outcomes are still lacking. Consequently, the overall level of evidence remains relatively low.

Several methodological limitations should also be considered. Observational studies are inherently susceptible to residual confounding, selection bias, and indication bias, particularly given the complex metabolic and CV comorbidity burden frequently observed in patients receiving GLP-1RAs. In addition, heterogeneity across studies in terms of patient populations, disease duration, background antirheumatic therapies, and definitions of disease activity makes direct comparison challenging.

Another important limitation is that improvements in inflammatory outcomes may partly reflect indirect metabolic effects, including weight loss and improved insulin sensitivity, rather than exclusively direct immunomodulatory mechanisms. Given the available study designs, it is not possible to disentangle whether the observed anti-inflammatory effects result from reduced adipose tissue-driven inflammation or from direct immunomodulatory actions of the therapeutic agents.

Furthermore, many currently available reports include relatively small sample sizes and short follow-up durations, limiting the ability to draw definitive conclusions regarding long-term efficacy and safety in RA populations.

## 5. Future Directions

Although preliminary findings appear encouraging, future research should address several critical gaps to better define the role of GLP-1RAs in rheumatoid arthritis. RCTs with adequate statistical power, specifically enrolling patients with RA, are needed to evaluate their effects on validated disease activity scores and imaging outcomes, moving beyond the current reliance on observational data. Mechanistic studies should further disentangle direct immunomodulatory effects from those secondary to metabolic improvements, clarifying the relative contribution of each pathway. Efforts to identify patient subgroups most likely to benefit—particularly individuals with obesity, insulin resistance, or distinct inflammatory profiles—will be essential to enable a more personalized therapeutic approach.

In addition, studies exploring combination strategies with c- or b-DMARDs are warranted to assess potential synergistic effects on both inflammation and cardiometabolic risk, as well as long-term safety. Given the high CV burden in RA, dedicated CVOTs in RA populations are also needed to determine whether the benefits observed in metabolic populations translate to this setting. Finally, the development of next-generation incretin-based therapies, including dual or multi-agonists, offers a promising avenue to enhance immunometabolism modulation and warrants further investigation in RA.

In conclusion, GLP-1RAs could represent a promising adjunctive therapeutic option in RA, particularly among patients with a high cardiometabolic burden, by targeting both metabolic dysregulation and inflammatory pathways. These findings support a more personalized medicine approach, where therapeutic decisions are guided by an integrated assessment of disease activity, metabolic status, and CV risk. Accordingly, optimal care may require a coordinated multidisciplinary model involving rheumatology, endocrinology, and cardiology expertise. Nonetheless, the current evidence in RA remains predominantly observational and heterogeneous, precluding definitive conclusions regarding efficacy and causality. Well-designed RCTs are therefore urgently needed to clarify the therapeutic role and long-term impact of GLP-1RAs in RA management.

## Figures and Tables

**Table 1:** Summary of preclinical experimental studies evaluating the effects of glucagon-like peptide-1 receptor agonists (GLP-1RAs) in rheumatoid arthritis. Abbreviations: RA: rheumatoid arthritis; RA-FLSs: rheumatoid arthritis fibroblast-like synoviocytes; TNF-α: tumor necrosis factor alpha; IL: interleukin; MCP-1: monocyte chemoattractant protein-1; HMGB-1: high mobility group box 1; MMP: matrix metalloproteinase; ROS: reactive oxygen species; NOX-4: NADPH oxidase 4; GSH: glutathione; 4-HNE: 4-hydroxynonenal; JNK: c-Jun N-terminal kinase; NF-κB: nuclear factor kappa B; MAPK: mitogen-activated protein kinase; AP-1: activator protein 1; ↓: reduction; ↑: increase.

Author (Year)	Study Design	Model	GLP-1RA	Main Outcomes	
				Oxidative Stress	Inflammatory Pathways
ZHENG ET AL. (2019) [15]	In vitro	TNF-stimulated human RA-FLSs	Dulaglutide	- Restored mitochondrial membrane potential; - ↓ ROS and NOX-4; - ↑ intracellular GSH.	- ↓ IL-1β, IL-6, MCP-1, HMGB-1; - ↓ MMP-3 and MMP-13; - ↓ JNK phosphorylation and NF-κB activation.
TAO ET AL. (2024) [16]	In vitro	TNF-stimulated human RA-FLSs	Exenatide (exendin-4)	- Improved mitochondrial function; - ↓ ROS and NOX-4; - restoration of GSH.	- ↓ IL-1β, IL-6, MCP-1, HMGB-1; - ↓ MMP-3 and MMP-13; - ↓ p38/MAPK and NF-κB signaling.
DU ET AL. (2019) [17]	In vitro	IL-1β-stimulated human RA-FLSs	Lixisenatide	- ↓ ROS and 4-HNE; - Prevention of apoptosis.	- ↓ TNF-α, IL-6, IL-8; ↓ MMP-1, MMP-3, MMP-13. - ↓ JNK, AP-1 and NF-κB signaling.

**Table 2:** Summary of clinical studies evaluating the effects of glucagon-like peptide-1 receptor agonists (GLP-1RAs) in rheumatoid arthritis. Abbreviations: RA: rheumatoid arthritis; T2DM: type 2 diabetes mellitus; PsA: psoriatic arthritis; GLP-1RA: glucagon-like peptide-1 receptor agonist; DAS28: Disease Activity Score in 28 joints; EULAR: European Alliance of Associations for Rheumatology; HbA1c: glycated hemoglobin; SJ: swollen joint; WL: weight loss; CRP: C-reactive protein; ESR: erythrocyte sedimentation rate; BMI: body mass index; VAS: visual analog scale; BW: body weight; ↓: reduction.

Author (Year)	Study Design	Population	GLP-1RA	Main Outcomes	
				Metabolic	Inflammatory
SULLIVAN ET AL. (2013) [18]	Prospective observational	11 RA and 4 PsA patients with T2DM	Liraglutide	- ↓ BW in DAS28 responders; - ↓ HbA1c in DAS28 responders.	- DAS28 reduction (4.2→2.7) in responders; - 9/15 achieved EULAR response; - ↓ SJ count in DAS28 responders.
GAVAZOVA ET AL. (2024) [19]	Observational	30 patients with RA, obesity and T2DM	Unspecified	- ~10% WL.	- ↓ Morning stiffness; - ↓ Joint swelling; - ↓ Finger pain; - ↓ CRP and ESR.
STIPHO ET AL. (2024) [20]	Retrospective observational (TriNetX database)	12,139 RA patients	Semaglutide	−	- ↓ Synovitis; - ↓ Stiffness; - ↓ Pain; - ↓ Swelling; at 30, 90 days, and 1 year.
DENTE ET AL. (2024) [21]	Retrospective observational	152 RA patients with obesity	Semaglutide, tirzepatide	- ↓ BW; - ↓ BMI; - ↓ Cholesterol and triglycerides.	- ↓ ESR and CRP; - ↓ VAS pain.
KELLNER ET AL. (2025) [22]	Retrospective observational	173 RA patients with BMI ≥27 and 42 controls	Semaglutide, tirzepatide	- ↓ Weight; - ↓ Cholesterol and triglycerides; - ↓ HbA1c.	- ↓ RA disease activity; - ↓ ESR and CRP; - ↓ VAS pain.

**Table 3:** Summary of cardiovascular outcomes associated with GLP-1 receptor agonists in patients with rheumatoid arthritis. Abbreviations: RA: rheumatoid arthritis; GLP-1RA: glucagon-like peptide-1 receptor agonist; T2DM: type 2 diabetes mellitus; DPP-4: dipeptidyl peptidase-4; JAKi: Janus kinase inhibitors; BMI: body mass index; HbA1c: glycated hemoglobin; LDL-C: low-density lipoprotein cholesterol; ESR: erythrocyte sedimentation rate; CRP: C-reactive protein; BP: blood pressure; CV: cardiovascular; TE: thromboembolic; MI: myocardial infarction; DVT: deep vein thrombosis; PE: pulmonary embolism; HR: hazard ratio; RR: risk ratio; BW: body weight; ↓: reduction.

Author (Year)	Study Design	Population	Intervention	Main Outcomes in the GLP-1RA Group	Key Limitations
KELLNER ET AL. (2025) [22]	Retrospective observational	173 RA patients with BMI ≥27 and 42 controls	GLP-1RAs (semaglutide or tirzepatide) vs. No therapy	- ↓ BW - ↓ BMI, - ↓ HbA1c, - ↓ Total cholesterol; - ↓ LDL-C and triglycerides, - ↓ ESR, and CRP; - no significant BP change.	- Retrospective design; - small sample size; - single center; - no evaluation of MACEs.
WANG AND ANTHONY (2025) [66]	Propensity score–matched cohort	8697 RA patients with T2DM	GLP-1RAs vs. DPP-4 inhibitors	- ↓ Risk of TE events (HR 0.76); MI (HR 0.72), cerebral infarction (HR 0.76), and DVT (HR 0.70); - ↓ arterial (HR 0.74) and venous thrombosis (HR 0.80); - ↓ all-cause mortality (HR 0.56); - PE not significantly different.	- Observational design; - no stratification by individual GLP-1RA agents.
BELTAGY ET AL. (2025) [67]	Propensity score–matched cohort	RA patients receiving JAKi	GLP-1RAs added to JAKi vs. JAKi alone	- ↓ Arterial CV events (5.3% vs. 7.9%; RR 0.673) and acute coronary syndromes (RR 0.645); - ↓ DVT and PE (RR 0.69); - Non-significant reductions in cerebral infarction and peripheral arterial thrombosis; - No difference in overall survival.	- Abstract data; observational design; - limited methodological details; - no stratification by specific GLP-1RA.

## Data Availability

No new data were created or analyzed in this study. Data sharing is not applicable to this article.

## Supplementary Materials

The following supporting information can be downloaded at: https://www.mdpi.com/article/10.3390/jpm16060284/s1, Supplementary Material S1: Complete search strategies; Table S1: Preferred Reporting Items for Systematic reviews and Meta-Analyses extension for Scoping Reviews (PRISMA-ScR) Checklist; Table S2: Summary of major clinical trials and meta-analyses assessing glucagon-like peptide-1 (GLP 1) receptor agonists on type 2 diabetes mellitus; Table S3: Summary of major clinical trials and meta-analyses assessing glucagon-like peptide-1 (GLP 1) receptor agonists on obesity; Table S4: Summary of major clinical trials and meta-analyses assessing glucagon-like peptide-1 (GLP 1) receptor agonists on cardiovascular comorbidities.
