# Supplementary material for: Glucagon-like Peptide-1 Receptor Agonists in Rheumatoid Arthritis: A Scoping Review of Metabolic, Anti-Inflammatory, and Cardioprotective Effects
Source: J Pers Med. 2026 May 26;16(6):284. doi: 10.3390/jpm16060284 (PMC13300951; doi:10.3390/jpm16060284)
Supplement: Supplementary file 1 [file jpm-16-00284-s001.zip › jpm-4282315-supplementary.pdf]

Supplementary material

Supplementary Material S1. Complete search strategies.

((Glucagon-Like Peptide-1 Receptor [Title/Abstract]) OR (GLP-1RAs[Title/Abstract]) OR (Dulaglutide[Title/Abstract]) OR (Exenatide[Title/Abstract]) OR (Lixisenatide[Title/Abstract]) OR (Liraglutide[Title/Abstract]) OR (Semaglutide[Title/Abstract]) OR (tirzepatide[Title/Abstract])) AND ((Rheumatoid Arthritis[Title/Abstract]) OR (inflammatory arthritis [Title/Abstract])).

((Glucagon-Like Peptide-1 Receptor [Title]) OR (GLP-1RA[Title]) OR (exenatide[Title]) OR (dulaglutide[Title]) OR (liraglutide[Title]) OR (lixisenatide[Title]) OR (semaglutide[Title]) OR (tirzepatide[Title]) OR (cagrisema[Title]) OR (retatrutide[Title]) OR (orforglipron[Title])) AND ((Diabetes[Title]) OR (glycemic control [Title])).

((Glucagon-Like Peptide-1 Receptor [Title]) OR (GLP-1RA[Title]) OR (exenatide[Title]) OR (dulaglutide[Title]) OR (liraglutide[Title]) OR (lixisenatide[Title]) OR (semaglutide[Title]) OR (tirzepatide[Title]) OR (cagrisema[Title]) OR (retatrutide[Title]) OR (orforglipron[Title])) AND ((Obesity[Title]) OR (overweight[Title]) OR (weight loss[Title]) OR (weight management[Title]) OR (obese[Title])).

((Glucagon-Like Peptide-1 Receptor [Title]) OR (GLP-1RA[Title]) OR (exenatide[Title]) OR (dulaglutide[Title]) OR (liraglutide[Title]) OR (lixisenatide[Title]) OR (semaglutide[Title]) OR (tirzepatide[Title]) OR (cagrisema[Title]) OR (retatrutide[Title]) OR (orforglipron[Title])) AND ((blood pressure[Title]) OR (hypertension[Title])).

((Glucagon-Like Peptide-1 Receptor [Title]) OR (GLP-1RA[Title]) OR (exenatide[Title]) OR (dulaglutide[Title]) OR (liraglutide[Title]) OR (lixisenatide[Title]) OR (semaglutide[Title]) OR (tirzepatide[Title]) OR (cagrisema[Title]) OR (retatrutide[Title]) OR (orforglipron[Title])) AND ((lipid*[Title]) OR (hypercholesterol* [Title]) OR (hypertriglycerid*[Title]) OR (HDL[Title]) OR (LDL[Title]) OR (lipoprotein[Title]) OR (cholesterol[Title]) OR (triglycerides[Title])).

((Glucagon-Like Peptide-1 Receptor [Title]) OR (GLP-1RA[Title]) OR (exenatide[Title]) OR (dulaglutide[Title]) OR (liraglutide[Title]) OR (lixisenatide[Title]) OR (semaglutide[Title]) OR (tirzepatide[Title]) OR (cagrisema[Title]) OR (retatrutide[Title]) OR (orforglipron[Title])) AND ((atheroscler*[Title]) OR (plaque[Title])).

((Glucagon-Like Peptide-1 Receptor [Title]) OR (GLP-1RA[Title]) OR (exenatide[Title]) OR (dulaglutide[Title]) OR (liraglutide[Title]) OR (lixisenatide[Title]) OR (semaglutide[Title]) OR (tirzepatide[Title]) OR (cagrisema[Title]) OR (retatrutide[Title]) OR (orforglipron[Title])) AND ((Cardiovascular[Title]) OR (stroke[Title]) OR (embol*[Title]) OR (thrombo*[Title]) OR (cardiac[Title]) OR (cerebrovascular[Title]) OR (myocardial infarction[Title])).

Supplementary Table S1. **Preferred Reporting Items for Systematic reviews and Meta-Analyses extension for Scoping Reviews (PRISMA-ScR) Checklist**

SECTION	ITEM	PRISMA-ScR CHECKLIST ITEM	REPORTED ON PAGE #
**TITLE**			
Title	1	Identify the report as a scoping review.	1
**ABSTRACT**			
Structured summary	2	Provide a structured summary that includes (as applicable): background, objectives, eligibility criteria, sources of evidence, charting methods, results, and conclusions that relate to the review questions and objectives.	1
**INTRODUCTION**			
Rationale	3	Describe the rationale for the review in the context of what is already known. Explain why the review questions/objectives lend themselves to a scoping review approach.	2
Objectives	4	Provide an explicit statement of the questions and objectives being addressed with reference to their key elements (e.g., population or participants, concepts, and context) or other relevant key elements used to conceptualize the review questions and/or objectives.	2
**METHODS**			
Protocol and registration	5	Indicate whether a review protocol exists; state if and where it can be accessed (e.g., a Web address); and if available, provide registration information, including the registration number.	3
Eligibility criteria	6	Specify characteristics of the sources of evidence used as eligibility criteria (e.g., years considered, language, and publication status), and provide a rationale.	3
Information sources*	7	Describe all information sources in the search (e.g., databases with dates of coverage and contact with authors to identify additional sources), as well as the date the most recent search was executed.	3
Search	8	Present the full electronic search strategy for at least 1 database, including any limits used, such that it could be repeated.	3
Selection of sources of evidence†	9	State the process for selecting sources of evidence (i.e., screening and eligibility) included in the scoping review.	3
Data charting process‡	10	Describe the methods of charting data from the included sources of evidence (e.g., calibrated forms or forms that have been tested by the team before their use, and whether data charting was done independently or in duplicate) and any processes for obtaining and confirming data from investigators.	3
Data items	11	List and define all variables for which data were sought and any assumptions and simplifications made.	3
Critical appraisal of individual sources of evidence§	12	If done, provide a rationale for conducting a critical appraisal of included sources of evidence; describe the methods used and how this information was used in any data synthesis (if appropriate).	3
Synthesis of results	13	Describe the methods of handling and summarizing the data that were charted.	3
**RESULTS**			

SECTION	ITEM	PRISMA-ScR CHECKLIST ITEM	REPORTED ON PAGE #
Selection of sources of evidence	14	Give numbers of sources of evidence screened, assessed for eligibility, and included in the review, with reasons for exclusions at each stage, ideally using a flow diagram.	Supplementary figure S1
Characteristics of sources of evidence	15	For each source of evidence, present characteristics for which data were charted and provide the citations.	3-12
Critical appraisal within sources of evidence	16	If done, present data on critical appraisal of included sources of evidence (see item 12).	3-12
Results of individual sources of evidence	17	For each included source of evidence, present the relevant data that were charted that relate to the review questions and objectives.	3-12
Synthesis of results	18	Summarize and/or present the charting results as they relate to the review questions and objectives.	3-12
**DISCUSSION**			
Summary of evidence	19	Summarize the main results (including an overview of concepts, themes, and types of evidence available), link to the review questions and objectives, and consider the relevance to key groups.	12-14
Limitations	20	Discuss the limitations of the scoping review process.	12-14
Conclusions	21	Provide a general interpretation of the results with respect to the review questions and objectives, as well as potential implications and/or next steps.	12-15
**FUNDING**			
Funding	22	Describe sources of funding for the included sources of evidence, as well as sources of funding for the scoping review. Describe the role of the funders of the scoping review.	15

JBI = Joanna Briggs Institute; PRISMA-ScR = Preferred Reporting Items for Systematic reviews and Meta-Analyses extension for Scoping Reviews.

* Where *sources of evidence* (see second footnote) are compiled from, such as bibliographic databases, social media platforms, and Web sites.

† A more inclusive/heterogeneous term used to account for the different types of evidence or data sources (e.g., quantitative and/or qualitative research, expert opinion, and policy documents) that may be eligible in a scoping review as opposed to only studies. This is not to be confused with *information sources* (see first footnote).

‡ The frameworks by Arksey and O'Malley (6) and Levac and colleagues (7) and the JBI guidance (4, 5) refer to the process of data extraction in a scoping review as data charting.

§ The process of systematically examining research evidence to assess its validity, results, and relevance before using it to inform a decision. This term is used for items 12 and 19 instead of "risk of bias" (which is more applicable to systematic reviews of interventions) to include and acknowledge the various sources of evidence that may be used in a scoping review (e.g., quantitative and/or qualitative research, expert opinion, and policy document).

From: Tricco AC, Lillie E, Zarin W, O'Brien KK, Colquhoun H, Levac D, et al. PRISMA Extension for Scoping Reviews (PRISMAScR): Checklist and Explanation. Ann Intern Med. 2018;169:467–473. doi: 10.7326/M18-0850.

Supplementary Table S2. Summary of major clinical trials and meta-analyses assessing glucagon-like peptide-1 (GLP-1) receptor agonists on type 2 diabetes mellitus. *Abbreviations:*
RCT = randomized controlled trial; duration (wk/wks) = study duration in weeks; **T2DM** = type 2 diabetes mellitus; **OADs** = oral antidiabetic drugs; **QW** = once weekly; **BID** = twice daily; **QD** = once daily; **TID** = three times daily; **PBO** = placebo; **ER** = extended-release; **SC** = subcutaneous; HbA1c = glycated hemoglobin; **FPG/FSG** = fasting plasma/serum glucose; **PPG** = postprandial glucose; **SMPG/SMBG** = self-measured plasma/blood glucose; **CGM** = continuous glucose monitoring; **BP** = blood pressure; **pts** = patients; **NI** = non-inferiority; **Δ** = treatment difference; **ETD** = estimated treatment difference; **CV** = cardiovascular; **DB** = double-blind; ↓ = significant decrease; ↑ = significant increase; ↔ = no significant change; ≈ = approximately equal to.

	**AUTHOR (YEAR)**	**STUDY DESIGN**	**POPULATION**	**GLP-1RA / DOSE**	**MAIN OUTCOMES**
**TYPE 2 DIABETES MELLITUS**	***Drucker et al. (2008)* [28]**	RCT, open-label, active-comparator DURATION	T2DM (drug-naïve or on OADs)	Exenatide 2 mg QW *vs* Exenatide 10 µg BID	QW: ↓HbA1c (−1.9% *vs* −1.5%), ↑pts HbA1c ≤7% (77% vs 61%), ↓FPG; both: ↓PPG (greater ↓PPG with BID)
	Wysham et al. (2014) [29]	RCT, 52 wk, multicenter AWARD-1	T2DM	Dulaglutide 1.5 mg QW, Dulaglutide 0.75 mg QW, Exenatide 10 µg BID, PBO	HbA1c ↓: 1.5>0.75>Exe>PBO; HbA1c %<7:1.5>0.75>Exe>PBO; FSG ↓: 1.5>0.75>Exe>PBO; PPG ↓: Dula>Exe/PBO; Wt: 1.5↓≈Exe, 0.75≈0;
	Dungan et al. (2014) [30]	RCT, open-label, multicenter AWARD-6	T2DM	Dulaglutide 1.5 mg QW, Liraglutide 1.8 mg QD	HbA1c ↓: Dula −1.42%, Lira −1.36%, Δ −0.06% → NI; % <7% HbA1c: similar; FSG & PPG ↓: similar;
	Nauck et al. (2016) [31]	RCT, 26 wk, randomized, open-label, parallel-group	T2DM	Liraglutide 1.8 mg QD *vs* Lixisenatide 20 µg QD	HbA1c ↓: Lira > Lixi (−0.62%); HbA1c <7%: Lira > Lixi (74.2% *vs* 45.5%); HbA1c ≤6.5%: Lira > Lixi; FPG ↓: Lira > Lixi; SMPG (overall) ↓: Lira > Lixi; PPG: Lixi > Lira (post-injection meal only), ≈ overall;
	Sorli et al. (2017) [32]	Phase 3a RCT, 30 wk, randomized, double-blind, placebo-controlled, parallel-group SUSTAIN-1	T2DM	Semaglutide 0.5 mg QW, Semaglutide 1.0 mg QW *vs* PBO	HbA1c ↓: Sema (1.0 ≈ 0.5) >> PBO (−1.55%, −1.45% vs −0.02%); HbA1c targets: Sema > PBO; FPG ↓: Sema > PBO; PPG ↓: Sema > PBO;
	***Ahmann et al. (2018)* [33]**	Phase 3a RCT, 56 wk, randomized, open-label, parallel-group SUSTAIN-3	T2DM	Semaglutide 1.0 mg QW *vs* Exenatide ER 2.0 mg QW	HbA1c ↓: Sema > Exe ER (−0.62%); HbA1c <7%: Sema > Exe ER (67% vs 40%); HbA1c ≤6.5%: Sema > Exe ER; FPG ↓: Sema > Exe ER; SMPG ↓ (overall): Sema > Exe ER; PPG ↓: Sema > Exe ER (overall);
	***R. E. Pratley et al. (2018)* [34]**	Phase 3b RCT, 40 wk, randomized, open-label, parallel-group SUSTAIN-7	T2DM	Semaglutide 0.5 mg QW *vs* Dulaglutide 0.75 mg QW; Semaglutide 1.0 mg QW *vs*	HbA1c ↓: Sema > Dula (both doses; −0.40% low dose, −0.41% high dose); HbA1c <7%: Sema > Dula; HbA1c ≤6.5%: Sema > Dula; FPG ↓: Sema ≥ Dula (significantly greater at high

				Dulaglutide 1.5 mg QW	dose); SMBG ↓ (overall): Sema > Dula; PPG ↓: Sema > Dula (overall);
	Capehorn et al. ***(2020)* [35]**	Phase 3b RCT, 30 wk, randomized, open-label, parallel-group SUSTAIN-10	T2DM	Semaglutide 1.0 mg QW *vs* Liraglutide 1.2 mg QD	HbA1c ↓: Sema > Lira (−0.69%); HbA1c <7%: Sema > Lira (80% vs 46%); HbA1c ≤6.5%: Sema > Lira; FPG ↓: Sema > Lira; SMBG ↓ (overall): Sema > Lira; PPG ↓: Sema > Lira;
	Aroda et al. ***(2019)* [36]**	Phase 3a RCT, 26 wk, randomized, double-blind, placebo-controlled, parallel-group PIONEER-1	T2DM	Oral semaglutide 3 mg, 7 mg, 14 mg QD *vs* PBO	HbA1c ↓: dose-dependent, Sema > PBO (−0.6%, −0.9%, −1.1% treatment policy; −0.7%, −1.2%, −1.4% trial product); HbA1c (<7%, ≤6.5%): Sema > PBO (all doses); FPG ↓: Sema > PBO (all doses);
	R. Pratley et al. ***(2019)* [37]**	Phase 3a RCT, 52 wk, randomized, double-blind, PIONEER-4	T2DM	Oral semaglutide up to 14 mg QD *vs* SC liraglutide 1.8 mg QD *vs* PBO	HbA1c ↓: Sema ≥ Lira, Sema > PBO; HbA1c <7% and ≤6.5%: Sema ≥ Lira, Sema > PBO; FPG ↓: Sema > PBO, Sema ≥ Lira; SMBG ↓: Sema ≥ Lira, Sema > PBO
	Yamada et al. ***(2020)*** [38]	Phase 2/3a RCT, 52 wk, randomized, placebo- and active-controlled PIONEER-9	T2DM	Oral semaglutide 3, 7, 14 mg QD *vs* PBO QD *vs* SC Liraglutide 0.9 mg QD	HbA1c ↓ dose-dependent: Sema 14 mg (−1.7%), 7 mg (−1.5%), 3 mg (−1.1%) vs PBO (−0.1%); Sema 14 mg > Lira −1.4%; HbA1c <7% or ≤6.5%: Sema 14 mg > Lira, Sema 3, 7, 14 mg > PBO ;
	Yabe et al. ***(2020)* [39]**	Phase 3a RCT, 52 wk, open-label, active-controlled PIONEER-10	T2DM	Oral Semaglutide 3, 7, 14 mg QD *vs* SC Dulaglutide 0.75 mg QW	HbA1c ↓ dose-dependent: Sema 14 mg > Dula (−1.7% vs −1.4%), Sema 7 mg ≈ Dula, Sema 3 mg < Dula; HbA1c <7% or ≤6.5%: Sema 14 mg > Dula, Sema 7 mg ≈ Dula, Sema 3 mg < Dula;
	Rosenstock et al. ***(2021)* [43]**	Phase 3 RCT, 40 wks double-blind, placebo-controlled SURPASS-1	T2DM	Tirzepatide 5, 10, 15 mg QW *vs* placebo	HbA1c: ↓ (−1.87%, −1.89%, −2.07% *vs* +0.04%); ETD −1.91% to −2.11% HbA1c <7%: 87–92% *vs* 19–20%; ≤6.5%: 81–86% *vs* 10%; <5.7%: 31–52% *vs* 1%. FPG: ↓ (≈ −44 −49 mg/dL *vs* +13 mg/dL); PPG/SMBG: ↓ (pre- + post-prandial; <140 mg/dL achieved)
	Ludvik et al. ***(2021)* [40]**	Phase 3 RCT, 52 wks, open-label, active-comparator, multicentre SURPASS-3	T2DM	Tirzepatide 5, 10, 15 mg QW *vs* insulin glargine (titrated)	HbA1c: ↓ dose-dependent (−1.93, −2.20, −2.37% *vs* −1.34%); superiority *vs* degludec (ETD −0.59% to −1.04) HbA1c <7% 82–93% *vs* 61%
	Del Prato et al. ***(2021)* [41]**	Phase 3 RCT, 52 wks, open-label, active-comparator, multicentre SURPASS-4	T2DM with high CV risk	Tirzepatide 5, 10, 15 mg QW *vs* insulin glargine (titrated)	HbA1c: ↓ dose-dependent (−2.24, −2.43, −2.58% *vs* −1.44%); superiority *vs* glargine (Δ up to −1.14%) HbA1c <7%:81–91% *vs* 51%; ≤6.5% in 66–81% *vs* 32%; <5.7% in 23–43% vs 3%. FPG: ≈ reductions *vs* glargine SMBG: > reductions in pre- and

					post-prandial glucose *vs* glargine.
	Rosenstock, Frías, et al. (2023) **[42]**	Phase 3b RCT, 52wks,open-label, active-comparator SURPASS-6	T2DM	Tirzepatide 5, 10, 15 mg QW + glargine *vs* insulin lispro TID + glargine	HbA1c: ↓ (−2.1%; −1.9%, −2.2%, −2.3% *vs* −1.1%); ETD ≈ −0.79% to −1.13% → superiority. HbA1c <7%: 68% *vs* 36%; ≤6.5%: 56% *vs* 22%; <5.7%: 18% *vs* 3%. FPG**:** ↓ greater *vs* lispro. PPG/SMBG:↓ (≈/slightly > *vs* lispro).
	Jastreboff et al. (2025) **[44]**	Phase 3 RCT, double-blind, placebo-controlled (176 wks + 17-wk off-treatment)	Obesity + prediabetes	Tirzepatide 5, 10, 15 mg QW *vs* placebo	T2DM: ↓↓↓ incidence (1.3% *vs* 13.3%); sustained after off-Tx (2.4% *vs* 13.7%). Normoglycaemia: ↑ (≈90–93% vs 59%). Glycaemia (FPG/HbA1c): ↓ *vs* placebo. Overall: marked delay/prevention of T2DM progression.
	Frias, Hsia, et al. (2023) [45]	Phase 2 RCT, 26 wks, multicentre, double-blind	T2DM, HbA1c 7–10.5%, BMI ≥23 kg/m²	Orforglipron 3, 12, 24, 36, 45 mg/day; Dulaglutide 1.5 mg weekly; Placebo	HbA1c: −1.2 -2.1% *vs* PBO −0.43% *vs* dulaglutide −1.10% HbA1c <7%: 65–96 ≤6.5%: 45–84% Fasting glucose: up to −2.48 mmol/L
	Frias, Deenadayalan, et al. (2023) **[46]**	Phase 2 RCT, multicentre, , double-blind, active-controlled	T2DM, BMI ≥27 kg/m²	CagriSema (semaglutide 2.4 mg + cagrilintide 2.4 mg) QW; Semaglutide 2.4 mg QW; Cagrilintide 2.4 mg QW	HbA1c: −2.2% (CagriSema), −1.8% (semaglutide), −0.9% (cagrilintide); ETD vs cagrilintide −1.3%, vs semaglutide −0.4% FPG : −3.3 mmol/L (CagriSema), −2.5 (semaglutide), −1.7 (cagrilintide); CGM mean glucose ↓ Δ: CagriSema > semaglutide and > cagrilintide
	Rosenstock, Frias, et al. (2023) **[47]**	Phase 2 RCT, multicentre, , DB, placebo & active-controlled, parallel-group	T2DM, BMI 25–50 kg/m²	Retatrutide 0.5–12 mg QW Dulaglutide 1.5 mg QW, PBO	HbA1c ↓ dose-dependent: 0.5 mg ~0.4%, 4 mg ~1.3–1.4%, 8 mg ~1.9–2.0%, 12 mg ~2.0% FPG ↓ 4–12 mg: −1.2 to −3.8 mmol/L SMBG Δ: dose-dependent decrease, max −3.74 mmol/L in 12 mg

Supplementary Table S3. Summary of major clinical trials and meta-analyses assessing glucagon-like peptide-1 (GLP 1) receptor agonists on obesity. *Abbreviations:* **RCT** = Randomized Controlled Trial; **DB** = Double-Blind; **T2DM** = Type 2 Diabetes Mellitus; **QW** = Once Weekly; **QD** = Once Daily; **HbA1c** = Glycated Hemoglobin; **WC** = Waist Circumference; **BP** = Blood Pressure; **BMI** = Body Mass Index; **SGLT2i** = Sodium-Glucose Cotransporter-2 Inhibitors; ↓ = significant decrease; ↑ = significant increase; ≈ = approximately equal to.

	AUTHOR (YEAR)	STUDY DESIGN	POPULATION	GLP-1RA / DOSE	MAIN OUTCOMES
**OBESITY**	***Buse et al. (2010)* [50]**	RCT (DURATION-1)	T2DM	Exenatide QW	Weight ↓ 4.1–4.5 kg; 77–79%: HbA1c + weight ↓
	Pi-sunyer et al. (2015) 51	DB, placebo-controlled RCT	Obese (without diabetes)	Liraglutide 3.0 mg QD	Weight ↓ 8.4 kg; ≥5% in 63%, ≥10% in 33%; cardiometabolic improvement
	Garvey et al. (2022) [52]	Phase 3 RCT (STEP 5)	Obese/overweight	Semaglutide 2.4 mg QW	Weight ↓ 15.2%; ≥5% in 77%, ≥10% in 62%, ≥15% in 52%; improved WC, BP
	Wharton et al. (2025) [53]	Phase 3b RCT (STEP UP)	Obese	Semaglutide 7.2 mg QW	Weight ↓ 18.7%; dose-dependent ≥20% and ≥25%; waist ↓ 11.7 cm
	***Lingvay et al. (2025)* [54]**	Phase 3b RCT (STEP UP T2D)	Obese + T2DM	Semaglutide 7.2 mg QW	Weight ↓ 13.2%; WC ↓ 6.5 cm; HbA1c ↓ 1.5%
	Rosenstock et al. (2021) [43]	Phase 3 RCT (SURPASS-1)	T2DM	Tirzepatide 5–15 mg QW	Weight ↓ 7–9.5 kg; HbA1c ↓ 1.87–2.07%; glycemic targets achieved
	***Frias et al. (2023)* [55]**	Phase 3 RCT (SURMOUNT-2)	Obese + T2DM	Tirzepatide 10–15 mg QW	Weight ↓ 12.8–14.7%; ≥10% in up to 65%; HbA1c ↓≈ 2%; BMI ↓ ≈ 5 kg/m²
	***Jastreboff et al. (2023)* [56]**	Phase 2 RCT, double-blind, placebo-controlled	Obese or BMI 27-30 kg/m² + weight-related comorbidity)	Retatrutide 1-12 mg QW	Weight ↓: −8.7% (1 mg), −17.1% (4 mg), −22.8% (8 mg), −24.2% (12 mg), *vs* −2.1% (placebo); ≥15% weight loss: 60% (4mg), 75% (8mg), 83% (12mg) *vs* 2% (placebo)
	***Rosenstock et al.(2023)* [42]**	Phase 2 RCT, double-blind, placebo- and active-controlled	T2DM + Obese/overweight	Retatrutide 0.5–12 mg QW	Weight ↓: −3.2% (0.5 mg), −7.9% (4 mg escalation), −10.4% (4 mg), −16.8% (8 mg slow), −16.3% (8 mg fast), −16.9% (12 mg) *vs* 3.0% placebo and 2.0% dulaglutide; ≥15% weight loss significantly ↑ with doses ≥4 mg
	Frias et al. (2023) [45]	Phase 2 RCT, double-blind, multicenter	T2DM, diet/exercise ± metformin, BMI ≥23 kg/m²	Orforglipron 12–45 mg QD	Weight ↓ (mean): up to – 10.1 kg (Orforglipron); -3.9 (dulaglutide); - 2.2 (placebo)
	***Frias et al. (2023)* [46]**	Phase 2 RCT, double-blind, multicentre	T2DM + Obese/overweight on metformin ± SGLT2i	CagriSema 2.4 mg QW	Weight ↓: 15.6% (CagriSema) *vs* 5.1% (semaglutide) *vs* 8.1% (cagrilintide)

Supplementary Table S4. Summary of major clinical trials and meta-analyses assessing glucagon-like peptide-1 (GLP 1) receptor agonists on cardiovascular comorbidities. *Abbreviations*: **SBP** = Systolic Blood Pressure; **DBP** = Diastolic Blood Pressure; **GLP-1RA** = Glucagon-Like Peptide-1 Receptor Agonist; **RA** = Receptor Agonist; **GIP** = Glucose-Dependent Insulinotropic Polypeptide; **GCG** = Glucagon; **RR** = Relative Risk; **LDL-C** = Low-Density Lipoprotein Cholesterol; **HDL-C** = High-Density Lipoprotein Cholesterol; **TC** = Total Cholesterol; **TG** = Triglycerides; **T2D** = Type 2 Diabetes; **CTA** = Computed Tomography Angiography; **CV** = Cardiovascular; **DPP-4i** = Dipeptidyl Peptidase-4 Inhibitors; **FMD** = Flow-Mediated Dilation; **CIMT** = Carotid Intima-Media Thickness; **hs-CRP** = High-Sensitivity C-Reactive Protein; **PAI-1** = Plasminogen Activator Inhibitor-1; **ApoE–/–** = Apolipoprotein E Knockout (mouse model); **LDLr–/–** = Low-Density Lipoprotein Receptor Knockout (mouse model); **CVOTs** = Cardiovascular Outcome Trials; **MACE** = Major Adverse Cardiovascular Events; **MI** = Myocardial Infarction; **HF** = Heart Failure; **CKD** = Chronic Kidney Disease; **IRR** = Incidence Rate Ratio; ↓ = significant decrease; ↑ = significant increase; ↔ = no significant change.

	AUTHOR (YEAR)	STUDY DESIGN	POPULATION	GLP-1RA / DOSE	MAIN OUTCOMES
**BLOOD PRESSURE**	***Wong, Toh, et al., (2025)* [58]**	Meta-analysis (30 RCTs)	Obese/overweight	Exenatide Liraglutide Semaglutide	SBP ↓3.37 mmHg; DBP ↓1.05 mmHg. Semaglutide: SBP ↓3.96 / DBP ↓1.49. Liraglutide: SBP ↓2.72 / DBP ↓0.69. Exenatide: SBP ↓2.33.
	***Basile et al., (2025)* [57]**	Systematic review & meta-analysis (85 RCTs)	Obese/overweight	GLP-1RA dual RA (GIP/GLP-1) triple RA (GCG/GIP/GLP-1)	SBP ↓3.4 mmHg; DBP ↓0.9 mmHg. Dual: SBP ↓5.1 / DBP ↓1.8. Triple: SBP ↓6.6 / DBP ↓2.1. Mortality ↓18% (RR 0.82).
	***Chou et al., (2025)* [59]**	Systematic review & network meta-analysis (75 RCTs)	GLP1RAs treated patients	Semaglutide Liraglutide Dulaglutide Tirzepatide Retatrutide	Retatrutide: SBP ↓7.0 mmHg. Tirzepatide: SBP ↓5.2 / DBP ↓1.7. Semaglutide: SBP ↓3.4 / DBP ↓0.8.
**LIPID PROFILE**	***Sun et al. (2015)* [60]**	Systematic review & network meta-analysis (35 RCTs)	T2D	Exenatide Liraglutide Taspoglutide other GLP-1RAs	LDL-C ↓0.08–0.16 mmol/L; TC ↓0.16–0.27 mmol/L; TG ↓0.17–0.30 mmol/L (liraglutide/taspoglutide). HDL-C ↔ / slight ↓.
	***Yao et al. (2024)* [27]**	Network meta-analysis (15 GLP-1RAs)	T2D	Semaglutide Tirzepatide PEG-loxenatide ITCA-650 others	Semaglutide: LDL-C ↓0.16 mmol/L, TC ↓0.48 mmol/L. Tirzepatide: TG ↓0.89 mmol/L. ITCA-650: TG ↓1.59 mmol/L. PEG-loxenatide: HDL ↑0.16 mmol/L.
	***Rivera et al. (2024)* [61]**	Systematic review & network meta-analysis (26 phase-3 RCTs)	T2D	GLP-1RAs; Dual RA (GIP/GLP-1)	Dual RA: LDL-C ↓6.8–11.6%, TG ↓13.3–19.9%, TC ↓5.1–7.9%. GLP-1RAs: LDL-C ↓4.3–8.2%, TC ↓5.2–6.4%.
**ATHEROSCLEROSIS**	***Piotrowski et al. (2013)* [63]**	Observational cohort (CT angiography)	303 pts undergoing coronary CTA	Circulating endogenous GLP-1	Higher GLP-1 levels associated with ↑ coronary plaque burden (independent of CV risk factors)
	***Song et al. (2015)* [64]**	Meta-analysis (31 studies)	Patients with T2D (~60 yrs; BMI ~29 kg/m²)	Liraglutide Exenatide DPP-4i (Sitagliptin Vildagliptin) (*no stratification*)	FMD ↑ (acute studies); CIMT ↔ / slight ↓; inflammatory markers ↓ (hs-CRP, PAI-1); lipids ↓ (TC, LDL, TG).

	Rakipovski et al. (2018) **[62]**	Preclinical experimental study	ApoE−/− and LDLr−/− mice	Liraglutide; Semaglutide	Aortic plaque ↓; intima thickness ↓; vascular inflammation pathways ↓.
**MACE**	***Sattar et al. (2021)*** **[65]**	Updated systematic review & meta-analysis (8 CVOTs)	T2D and high CV risk	Lixisenatide Liraglutide Semaglutide Exenatide Albiglutide Dulaglutide Efpeglenatide	MACE ↓14%; CV death ↓; MI ↓; stroke ↓; all-cause mortality ↓12%; HF hospitalization ↓11%.
	Galli et al. (2025) **[25]**	Systematic review & meta-analysis (21 RCTs)	T2D ± Obesity, CKD, HF	Lixisenatide, Liraglutide, Exenatide, Semaglutide, Efpeglenatide, Dulaglutide, Albiglutide, Tirzepatide	All-cause mortality ↓12% (IRR 0.88); CV death ↓13% (IRR 0.87); MACE ↓13% (IRR 0.87); MI ↓15%; HF hospitalization ↓15%; stroke ↔
